# Staying in Place, Staying Connected: Exploring Tie-Retention to Inform Network-Based Health Interventions in Public Housing Communities

**DOI:** 10.1007/s11524-026-01122-x

**Published:** 2026-08-03

**Authors:** Mabeline Velez, Robert McDonough, Sharon M. Casey, Hye Jin Park, Gerardo Maupome, Raul I. Garcia, Neha Gondal, Brenda Heaton

**Affiliations:** Department of Health Policy & Health Services Research, Boston University Henry M. Goldman School of Dental Medicine, Boston, MA, USA; Department of Epidemiology, Boston University School of Public Health, Boston, MA, USA; Department of Health Policy & Health Services Research, Boston University Henry M. Goldman School of Dental Medicine, Boston, MA, USA; Department of Epidemiology, Boston University School of Public Health, Boston, MA, USA; University of Utah School of Dentistry, 530 Wakara Way, Salt Lake City, UT, USA; Department of Epidemiology, Richard M. Fairbanks School of Public Health, Indiana University, Indianapolis, IN, USA; Department of Health Policy & Health Services Research, Boston University Henry M. Goldman School of Dental Medicine, Boston, MA, USA; Department of Sociology, Boston University, Boston, MA, USA; Faculty of Computing & Data Sciences, Boston University, Boston, MA, USA; Department of Health Policy & Health Services Research, Boston University Henry M. Goldman School of Dental Medicine, Boston, MA, USA; Department of Epidemiology, Boston University School of Public Health, Boston, MA, USA; University of Utah School of Dentistry, 530 Wakara Way, Salt Lake City, UT, USA

**Keywords:** Social network analysis (MeSH), Public housing (MeSH), Natural disasters (MeSH), Health behavior (MeSH)

## Abstract

The COVID-19 pandemic disrupted human relationships through public health distancing mandates and migration shifts. These changes had the potential to be especially disruptive in public housing, which are government-funded developments for housing low-income, frequently mobile, populations. In light of these social upheavals, our goal is to leverage this natural experiment to understand the persistence of relationships, measured as ties in social networks, within disadvantaged public housing communities. We develop a predictive model of relationship maintenance, what we call ego-alter tie-retention, using data from an egocentric longitudinal survey of 187 adults residing in two Boston public-housing developments. Baseline data were collected between March 2019 and 2020. Our outcome variable is tie-retention (yes vs. no) of each reported tie at approximately 1 year post baseline. Predictors include the degree to which different types of tie overlap (multiplexity), tie-strength measures (frequency of contact, shared meals, closeness, years known, relationship type, residence), individual ego and alter attributes (age, education, employment, caregiver status), and homophily of relationships (based on gender, Hispanic ethnicity, housing category, caregiver status, sugar-sweetened beverage/food consumption, self-rated health, education, smoking). Multilevel hierarchical logistic regression with penalized quasi-likelihood estimation was used, and model fit was compared via Akaike information criterion. The best fitting model includes multiplexity, longer relationship duration, shared (i.e., homophilous) educational background, and ego-level smoking (AIC = 519.2). These findings have implications for the design of social network-based interventions in public housing contexts. Leveraging durable, homophilous social ties may achieve greater intervention longevity and reach in similar low-income urban settings.

## Introduction

Thesocial and physical contexts within which people live often shape their health behaviors and health outcomes through exposure to common risk factors and associated chronic disease outcomes [1, 2]. Given its social and physical context, public housing—a national model for housing the urban poor—creates a unique environment for considering health-promoting interventions that extend beyond the individual. Public housing residents are at greater risk of being exposed to conditions that adversely affect health. Poor health, in turn, reinforces poverty cycles by limiting employment opportunities. Thus, the relationship between health and poverty in public housing is reciprocal, warranting investigations into viable avenues for interventions.

A growing body of literature recognizes the importance of social network-based interventions that leverage an individual’s relationships with friends and family in modifying a variety of their health-related behaviors, including smoking cessation [3], weight reduction [4], physical activity [5], and overall well-being [6]. Effective social network-based interventions in public housing could improve health by reducing barriers associated with poor health and supporting pathways out of poverty [7, 8]. While social networks have been shown to promote behavioral modifications, whether this influence is sustained over time is not currently well known. There is a need for empirical research that focuses on the long-term structure and composition of social networks to determine the longer-term viability of network-based health promoting interventions, particularly among those populations at highest risk for poor health outcome [1].

Research on the evolution of social networks shows that a variety of sociodemographic attributes, such as age, gender, and educational attainment [9-13], influence the creation, retention, and dissolution of social network ties. The nature of relationships, or dyadic characteristics, also shapes social network stability. For instance, kinship ties are generally more stable than friendship ties [14]. Importantly, when sociodemographic and/or behavioral traits are shared between connected individuals (i.e., homophily), the likelihood of maintaining social ties over time tends to increase [9]. Another feature of close personal ties, multiplexity, is also related to tie evolution. Ties are multiplex if multiple kinds of relationships (e.g., sibling and neighbor) layer over each other. Multiplex ties are generally understood to be stronger [15, 16], meaning they are less prone to dissolution.

Despite these insights, scholarship on tie-retention within social networks based on longitudinal designs is limited, owing to the resource-intensive nature of tracking individuals and their networks over time [11, 17]. Furthermore, the slow evolution of social networks can draw out this demand for resources. The COVID-19 pandemic, however, served as a natural experiment by accelerating social network disruption through restrictions imposed on in-person interactions. The COVID-19 pandemic also generated unprecedented mobility [18], likely producing upheaval in interpersonal relationships. These disruptions accelerated social network changes that might otherwise have not occurred or may have taken years to occur, offering a unique opportunity to study tie dissolution and retention.

Public housing settings provide a unique context for studying network dynamics for multiple reasons. First, the COVID-19 pandemic disproportionately affected public housing residents with disparate consequences to health and economic prosperity [19]. Due to economic instability during the pandemic, public housing residents also experienced extensive job losses and housing insecurity [20, 21]. Second, attributable to the density of living conditions in public housing communities, residents’ networks tend to be locally clustered, and exhibit multiplexity (i.e., co-occurrence of multiple types of relationships), as well as homophily (i.e., the tendency for people who share characteristics and behaviors to form relationships with one another) [22]. These kinds of ties are a crucial source of social support for residents as they are known to play a vital role in childcare, healthcare access, and emotional or financial support [23-25]. The disproportionate burden of COVID-19 in public housing developments combined with dense and localized social networks means that studying network dissolution and retention in this context is especially important.

Yet, there is a paucity of research on how social networks in public housing communities tend to evolve over time. This lack of evidence poses significant challenges to the design of effective network-based health interventions. Interventions targeting ties that tend to be transient, for example, are unlikely to be successful in the long run. The goal of this study was to leverage the natural experiment born out of the COVID-19 pandemic to develop a predictive model of tie-retention among public housing residents. Specifically, the study examined how individual-level characteristics (for both participants/egos and named contacts/alters), together with homophily and tie-strength, contribute to the likelihood of maintaining social connections over time. We discuss implications of our findings for designing effective long-term social network health promotion interventions.

## Methods

### Study Population, Participant Recruitment, and Data Collection

We collected egocentric (i.e., ego or participant reported) longitudinal data from adults residing in two government-funded public housing developments situated in Boston, MA. Eligible individuals included those aged at least 18 years, who spoke English and/or Spanish, and planned to remain living in the public housing development for at least 3 years after enrollment. Participant recruitment occurred between March 2019 and March 2020, and individuals were contacted approximately 1 year later (June 2021 to August 2022) for a follow-up interview. Recruitment methods involved door-to-door visits and the distribution of study pamphlets for interested individuals to contact the study team. Interviews were conducted at the participant’s home or in a community space by three trained study personnel who were bilingual in English and Spanish [26]. Responses were entered directly into a REDCap data collection form [27].

At baseline and follow-up, egos (i.e., participants) were asked to complete several modules as part of a structured interview. Social networks were measured using three name generator questions where egos were asked to provide names of alters (i.e., social contacts) with whom they (1) discuss important matters, (2) share meals, and (3) regularly interact within the public housing communities. In addition, we captured (1) personal sociodemographic and health information, (2) ego perceptions of relationship attributes (i.e., closeness, length of time known) and sociodemographic and health history characteristics for each alter, and (3) reasons for relationships listed under each network generator (i.e., important matters, shared meals). Egos who completed both the baseline and follow-up interview were included in the analysis (*n* = 187). Egos who did not report any alters (*n* = 5) and/or who did not complete a follow-up survey (*n* = 85) were excluded from analysis (see Appendix Table 4).

### Dependent Variable

The primary outcome of this study was the retention of social network ties (yes vs no) at follow-up, approximately 1 year post baseline interview. At both baseline and follow-up, egos were asked to name individuals they had interacted with in the past year, using the following prompt: “Thinking about the past year, please tell me the names [first and last, or nickname] of the adults with whom you discussed important matters or from whom you sought advice. These may include people such as friends, family members, a spouse, or a significant other.” Relationships or ties were classified as retained at follow-up if egos mentioned the same alter at both the baseline and follow-up interview. Conversely, if an ego mentioned an alter at baseline but did not mention them again at follow-up, the tie was classified as lost (i.e., not retained).

### Independent Variables

#### Ego and Alter Individual-Level Characteristics

Egos reported on both personal and alter-level sociodemographic characteristics, including age (years), gender (female, male), race/ethnicity (White/non-Hispanic, Black/non-Hispanic, Hispanic, non-Hispanic), education level (no formal education, less than high school, GED/high school diploma, some/completed vocational/technical school, some/completed college, graduate/advanced degree), employment status (full-time, part-time, occasional, unemployed/student/homemaker, retired), and current caregiver status to children under 6 years (yes, no).

#### Relational/Tie-Strength Variables

Ego-alter relational attributes were assessed by asking egos to report on various tie-strength measures including the frequency of shared meals (“*In the past year, how often did you share meals or purchase food with or for [alter]?*”) and frequency of contact (“*In the past year, how often did you see or talk to [alter]?*”) Responses to both included ‘Daily’, ‘Weekly,’ ‘Less than every week,’ ‘Rarely or never.’ For analysis, these responses collapsed into ‘Daily’ versus ‘Less than daily.’ Egos reported on closeness of the relationship by responding to the following question: “*On a scale of 1 to 5, how close would you consider yourself to be to [alter’s name]*?” Responses ranged from ‘Not at all close’ to ‘Very close’ and were collapsed for analysis to ‘Very close’ as compared to all other responses. Egos reported the total number of years they had known each alter, with fourteen years being the threshold for the binary variable (“years known”), as this represented the average length of time that participants lived in their current public housing development. By using the average duration of the relationship as the cut point, this would likely differentiate between family versus other relationships. Relationships were dichotomized as ‘Friend/Other’ (including acquaintance, coworker or associate, educator/teacher, religious leader/mentor) and ‘Family’ (spouse/romantic partner, parent, sibling, child, other relative) for analysis.

#### Behavior, Health Status, and Homophily Variables

Self-reported and perceived status of alters on behavioral characteristics and health history were assessed. Egos self-reported their sugar-sweetened beverage (SSB) and sugar-sweetened food (SSF) consumption patterns (“How often do you drink sweet or sugary drinks (for example: juice, soda, pop, lemonade, Coke, Pepsi, Mountain Dew, Kool-Aid, Gatorade)?”; “How often do you eat sweet or sugary foods (for example: candy, cookies, donuts, ice-cream)?”. Egos provided their perceptions of alters’ SSB and SSF intake patterns. Responses were categorized as ‘Rarely or never,’ ‘Once a week,’ and ‘Daily,’ and further collapsed to ‘Daily’ and ‘Less than Daily’ for analytic purposes. Cigarette smoking behavior of egos and alters was classified as ‘Daily,’ ‘Non-daily,’ ‘Former,’ and ‘Never smoker,’ with daily smoking defined as smoking at least once daily, while a non-daily smoker was defined as someone who smokes from time to time. Responses were categorized as ‘Ever (current/former)’ and ‘Never’ for analysis.

Egos self-reported their oral health status and indicated their perception of each alter’s oral health status (“*Overall, how would you rate the health of your/[named alter’s] teeth and gums…?*”). Self- and ego-reported general health status was also assessed by asking, “*Would you say your/[named alter’s] health in general is…*”. Response options for both questions asking about oral health and general health status included ‘Excellent,’ ‘Very Good,’ ‘Good,’ ‘Fair,’ or ‘Poor’, and were further collapsed to ‘Excellent/Very Good/Good’ to ‘Fair/Poor’ for analysis.

For these same behaviors (i.e., frequency of SSB and SSF consumption, cigarette smoking) and health status measures (i.e., oral and general health status), we determined ego-alter homophily (i.e., the tendency for individuals to form relationships with individuals who are similar to them in terms of certain attributes and behaviors). If the ego’s self-reported response matched their report of each alter’s behavior, the ego-alter connection was determined to be homophilous on that trait, whereas discordant responses were classified as non-homophilous.

#### Network Tie-Type Predictors: Multiplexity

Multiplexity of social network ties (i.e., the extent to which different types of social ties layer over each other) was operationalized as the number of connections egos had with each alter under three specific domains. As described above, egos reported on alters with whom they (1) discuss important matters, (2) share meals, and/or (3) interact with in the public housing development. If an ego named an alter for any of the three prompts, the variable was labeled as a ‘1’ for that prompt, and ‘0’ otherwise. If an ego named an alter in only one of the three domains, the tie was treated as ‘non-multiplex’ or uniplex. If an alter was named in two domains, it was treated as having a multiplex value of ‘2,’ and if an alter was named for all three domains, it was treated as having a multiplex value of ‘3.’ We compared multiplex ties (multiplex = 2 or 3) with non-multiplex ties (multiplex = 1) to test if alters named in more domains of social interaction were more likely to be retained.

### Statistical Analysis

We tabulated descriptive statistics of ego and alter sociodemographic, health history, and behavioral characteristics. We also calculated bivariate associations (odds ratio (OR), 95% confidence intervals (CIs)) between independent variables and the outcome of retention of social network ties (yes vs no) at follow-up. These measures included network tie-type (i.e., multiplexity), ego-alter relational measures (i.e., frequency of shared meals, frequency of contact, closeness), ego-level and alter-level individual variables (i.e., age, education, employment, caregiver status), and ego-alter homophily measures (i.e., gender, Hispanic ethnicity, housing category, caregiver status, SSB and SSF consumption, and health status).

Multilevel (hierarchical) logistic regression with penalized quasi-likelihood estimation was used to model the association between independent variables (i.e., multiplexity, individual-level variables, tie-strength measures, and ego-alter homophily variables) and the outcome of tie-retention (yes vs no) at follow-up. As alters are connected to egos (as well as other alters), it is likely their behaviors are related to egos. To account for this interdependence, we modeled alters as nested within egos using a multilevel framework [19].

In building our regression models, we adopted an approach based on theory as well as forward-selection. Theoretically, research shows that strength of social network ties is a strong predictor of tie-retention [9, 28, 29]. We have several variables to capture tie strength. Our bivariate results (Table 3) showed that all measures of relationship closeness were strongly related to likelihood of tie-retention. However, as all these variables measure the same underlying concept of relational strength, they tend to be correlated. After running multivariable analyses including combinations of all these variables, we decided to use ‘years known’ between egos and alters as the variable that mostly closely approximates tie strength. While ‘closeness’ is also theoretically a strong predictor of tie-retention, we excluded this variable from our final model as the distribution of ‘closeness’ captures less variability than ‘years known.’ Subsequently, we followed a forward-selection approach, starting with multiplexity, followed by relational variables, and subsequently examining homophily variables, as these were found to be significantly associated with tie-retention in the bivariate analyses.

## Results

At baseline, a total of 628 ego-alter ties were reported by 187 egos/participants who were present at both the baseline and follow-up interview. Average network density, calculated as the percentage of alter-alter ties divided by the number of possible ties within the ego network, was 0.525. Table 1 reports baseline ego self-reported characteristics. Egos were, on average, aged 53 (Standard deviation (SD) = 13.6) years, the majority were female (*n* = 162, 86.6%), and most identified as Hispanic (*n* = 129, 69.7%). Most ego-alter ties were between family members (*n* = 342, 54.5%). About half of egos reported their general health and oral health status as ‘excellent/very good/good’ (57.8% and 50.3%, respectively). Approximately 72% of egos reported daily consumption of sugar-sweetened beverages (SSBs), 25.7% reported daily consumption of sugar-sweetened foods (SSFs), and the majority reported having never smoked (61%).

Table 2 presents data on alter-level, relational, and homophily measures at baseline, as reported by egos, both overall and stratified by retention of the ego-alter tie at follow-up (yes vs no). Of the 628 ego-alter ties at baseline, 79.5% (*n* = 499) were retained at follow-up. Among ties that were retained at follow-up, alters were slightly younger (48.8 vs. 50.2 years) and more likely to be female (77.4% vs. 72.1%), compared to ties that were not retained. Retained ties were more likely to live in the same household as egos (13.4% vs. 3.9%), though less likely to reside in the same housing development (23.7% vs. 44.2%), as compared to ties not retained at follow-up. Retained ties were also more likely to be family members (60.3% vs. 31.8%). Comparing relational attributes of tie-retention as reported by egos, ties present at follow-up were more likely to be ‘very close’ (84.4% vs. 58.1%), report daily contact (70.7% vs. 54.3%), and share daily meals (21.6% vs. 12.4%). Retained ties were less likely to be frequent smokers, as compared to lost ties (11% vs. 19.4%). Distributions of ego-reported SSB and SSF consumption frequency and health status of retained vs lost ties were generally similar. Ties that were homophilous on Hispanic ethnicity (95.8% vs. 86.1%) and education category (60.9% vs. 37.2%) were more likely to be retained. Overall, the proportion of ‘Don’t know’ or missing responses was infrequent.

Table 3 displays bivariate associations between network tie-type, including multiplexity, relational/tie-strength, individual-level (for both ego and alter), and homophily variables measured at baseline and the outcome of retention of ego-alter ties at follow-up (yes vs no). Network tie-type predictors were associated with the retention of ego-alter ties, including multiplexity (Multiplex = 2: OR = 1.9, 95% CI 1.02, 3.55; Multiplex = 3: OR = 4.43, 95% CI 1.84, 10.67). When examining relational measures, several tie-strength variables, including relationship type (OR = 4.2, 95% CI 2.37, 7.43), frequency of contact (OR = 2.63, 95% CI 1.43, 4.84), closeness (OR = 7.26, 95% CI 3.37, 15.62), and years known (OR = 5.59, 95% CI 2.86, 10.96), were associated with higher odds of tie-retention at follow-up. At the ego-level, the strongest associations were observed for employment status (> high school vs ≤ high school) (OR = 1.3, 95% CI 0.61, 2.81), Hispanic ethnicity (yes vs no) (OR = 1.46, 95% CI 0.64, 3.33), and daily consumption of SSBs (OR = 1.41, 95% CI 0.61, 3.27), although wide confidence intervals indicate that these estimates are imprecise. Ties that were homophilous on Hispanic ethnicity (OR = 3.18, 95% CI 1.23, 8.19), education category (OR = 2.89, 95% CI 1.68, 4.97), and caregiver status (OR = 2.14, 95% CI 1.08, 4.32) had higher odds of retaining ties at follow-up, compared to ties that were non-homophilous on these measures.

**Fig. 1:**
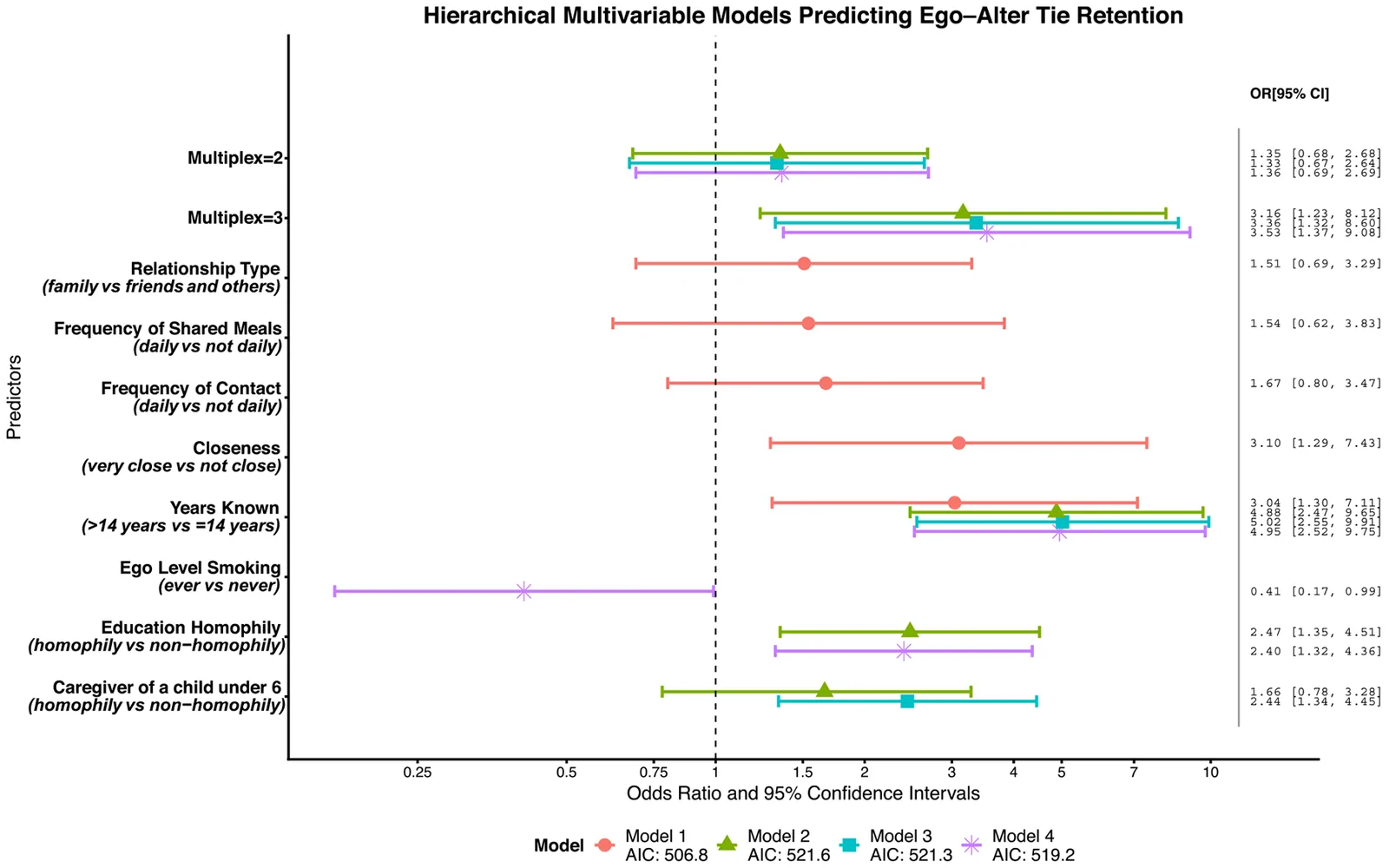
Hierarchical multivariable models predicting ego–alter tie retention. The figure shows odds ratios (ORs) and 95% confidence intervals from a series of multilevel logistic regression models estimating the likelihood that an ego–alter tie was retained at follow-up (yes vs. no) in this sample

Figure 1 shows the results of a series of multilevel logistic regression models predicting whether an ego-alter tie was retained at follow-up (yes vs no) in this sample. Model 1 contains several tie-strength variables, which are known predictors of tie-retention, including ‘relationship type,’ ‘frequency of shared meals,’ ‘frequency of contact,’ ‘closeness,’ and ‘years known.’ This model yielded a lower Akaike Information Criterion (AIC) value (AIC = 506.8) than the bivariate associations (Table 3). Yet, we observed a decrease in the magnitude of the association for each of the included relational variables as they are likely correlated with each other. While ‘closeness’ was a statistically significant predictor of tie-retention (OR = 3.1, 95% CI 1.30, 7.11) in this model, we removed it from subsequent models as its distribution was low relative to ‘years known.’ As such, in subsequent models, we retained only ‘years known’ as our key measure of tie-strength.

Model 2 included ‘years known’ as well as ‘multiplexity,’ ‘education homophily,’ and ‘caregiver status homophily’ (AIC = 521.6). This model shows that ‘years known’ (OR = 4.88, 95% CI 2.47, 9.65) and ‘education homophily’ (OR = 2.47, 95% CI 1.35, 4.51) are predictive of tie-retention, along with multiplex = 3 (OR = 3.16, 95% CI 1.23, 8.12). Multiplex = 2 is also no longer statistically significant, though maintains strength of association (OR = 1.35, 95% CI 0.68, 2.68). We note that ‘caregiver status homophily’ is no longer statistically significant (OR = 1.6, 95% CI 0.78, 3.28).

In Model 3, we removed ‘education homophily’ though we do not observe any gain in model fit (AIC = 521.3). Finally, in Model 4, we included ‘years known’ (OR = 4.95, 95% CI 2.52, 9.75), multiplexity’ (multiplexity = 2: OR = 1.36, 95% CI 0.69, 2.69; multiplexity = 3: OR = 3.53, 95% CI 1.37, 9.08), and ‘education homophily’ (OR = 2.47, 95% CI 1.32, 4.36) as statistically significant predictors of tie-retention. We also included ego-level smoking status (ever vs never) (OR = 0.41, 95% CI 0.17, 0.99) and removed ‘caregiver status homophily’ as it was no longer statistically significant. This resulted in the best fit model predicting tie-retention at follow-up (AIC = 519.2).

## Discussion

Our goal in this study was to investigate the retention of social network ties among residents of public housing communities in Boston, MA, treating the COVID-19 pandemic as a natural disruptor of relationships. Our results show that tie-retention is best explained by multiplexity of social ties, educational homophily, and relational closeness as captured by the duration of the relationship. Indeed, most retained ties were between people who had long-standing relationships, which aligns with prior research showing that ties that have existed for longer are more likely to remain stable even after disruptions [12]. At the same time, our findings are novel, as they pertain to neglected community-dwelling populations that are typically subject to long-term poverty.

Public housing residents often live in their homes for long periods of time, which makes for strong social bonds in the local community. Furthermore, common backgrounds and experiences, as we find in the case of education and caregiving responsibilities, may further reinforce these ties, making them more resilient even when external conditions change [12]. Our findings also show that multiplex relationships that serve multiple purposes, such as emotional support, shared meals, and resource exchange, are more likely to withstand the test of time and disruption. Burt have argued that multiplexity contributes to the stability of social ties, and our findings reinforce this idea, indicating that having multiple layers of connection helps sustain relationships over time [12]. While prior research has indicated that oral health and other health behaviors are important predictors of homophilous ties [1, 3-6], we did not observe homophily on health outcomes and behaviors to be predictors of tie-retention. One possible explanation is that relationships based on health and related behaviors may be weak ties formed more through shared need or information exchange than long-standing social connection, especially in contexts where both poverty and poor health are prevalent and intertwined [30, 31].

Our study has several strengths. To our knowledge, this is the first study to model tie-retention among low-income communities. Furthermore, existing research has primarily focused on specific age groups, leaving a gap in our understanding of how networks persist in disadvantaged settings. Additionally, the emergence of the COVID-19 pandemic in the middle of our original study period offered us an opportunity to study an otherwise understudied phenomenon in such communities—its effects on the severance and retention of close personal ties. Given that the COVID-19 pandemic disproportionately affected communities experiencing poverty, heightening financial burdens, and worsening health risks, including among public housing residents [32], our results likely reflect the minimally sufficient set of predictors under maximum disruption. More generally, our work contributes to the growing body of literature that examines factors influencing tie-retention over time. Importantly, we account for the multiplex nature of relationships, which is understood to be an important dimension of relationships [22] yet largely unexplored in tie dissolution and retention.

Our findings also have important implications for the design of network-based health interventions. Interventions aimed at generating changes in health-related behaviors are difficult not only because behavioral modifications can be challenging but also because of countervailing pressures from network members who exhibit similar behaviors, which can result in tie-dissolution. This makes it imperative that interventions target ties that can withstand pressures toward dissolution. Our findings suggest that interventions that leverage social bonds to instantiate changes in behaviors should focus on homophilous ties, those that are long-term, and those that are multiplex.

Our study also has some limitations. First, we relied on participants to remember the names of their contacts, which may introduce some error. To mitigate this, interviewers used structured recall prompts to facilitate participants’ memory of their prior reported social contacts. Another limitation is that many predictors in our model are highly correlated, making it difficult to isolate each variable’s independent effect. Although we accounted for these relationships, some interdependence may remain. However, we expect the impact to be limited to an inflation of the standard error, which would not meaningfully change our inferences. Future research could explore alternative methods—such as structural equation modeling or longitudinal designs—to better disentangle these complex associations. Finally, this study is limited to those who remained in the housing developments during the COVID-19 pandemic and were present for follow-up starting in May 2021; consequently, this group may not be a representative subset. However, those that left were less likely to be members of minority groups, were more educated, more likely to be employed, and self-reported better health status (see Appendix Table 4). Therefore, those who stayed in place were those most likely to be in greater need of health promoting interventions.

In conclusion, by highlighting how health and economic challenges enforce each other in public housing settings, our findings support the development of network-based interventions that are well-grounded in place and social connection, and that are tailor-made to the social connectivity features of the target group.

## Figures and Tables

**Table 1:** Baseline ego self-reported characteristics, March 2019–2020 (*N* = 187)

	*N*	%
**Age, years** *(mean, SD (range))*	52.6	13.6 (21, 90)
**Average network size (mean, SD (range))**		
**Gender—female**	162	86.63
**Race/ethnicity**		
White/non-Hispanic	2	1.08
Black/non-Hispanic	51	27.57
Hispanic	129	69.73
Non-Hispanic	3	1.62
**Education level**		
No formal education	2	1.07
Less than high school	73	39.04
GED/High school diploma	56	29.95
Some/completed vocational/technical school	9	4.81
Some/completed college	44	23.53
Graduate/advanced degree	2	1.07
**Employment status**		
Full-time job	34	18.18
Part-time job	55	29.41
Occasional job	5	2.67
Unemployed	72	38.5
Retired	21	11.23
**Primary caregiver of a child < 6 years (yes vs no)**	32	17.11
**Ego-alter relationship type** *		
Family	342	54.5
Friend and others	283	45.1
**General health status**		
Excellent	20	10.7
Very good	24	12.83
Good	64	34.22
Fair	67	35.83
Poor	12	6.42
**Oral health status**		
Excellent	21	11.23
Very good	16	8.56
Good	57	30.48
Fair	61	32.62
Poor	27	14.44
**Sugar-sweetened beverage consumption**		
Rarely or never	28	14.97
At least once a week, not every day	25	13.37
Once a day	44	23.53
Twice a day	34	18.18
Three times a day	26	13.9
Four times a day	13	6.95
Five or more times a day	17	9.09
**Sugar-sweetened food consumption**		
Rarely or never	78	41.71
At least once a week, not every day	61	32.62
Once a day	29	15.51
Twice a day	10	5.35
Three times a day	2	1.07
Four times a day	2	1.07
Five or more times a day	5	2.67
**Smoking status**		
Daily smoker	26	13.9
Non-daily smoker	8	4.28
Former smoker	39	20.86
Never smoked	114	60.96

*SD* standard deviation

* Family: spouse (current or former), romantic partner (current or former), parent (mother or father), sibling (brother or sister), another relative (e.g., uncle), and friend: friend, acquaintance, coworker or associate, health professional, and religious leader/mentor

**Table 2:** Baseline characteristics of alters, as reported by egos, March 2019–2020, stratified by retention of ego-alter ties at follow-up, June 2021–August 2022 (*N* = 628)

	Ego-alter ties at baseline		Retention of ego-alter ties at follow-up			
	*N* = 628		Yes (*N* = 499)		No (*N* = 129)	
	*N*	%	*N*	%	*N*	%
Alter-level characteristics						
**Average age, years (**SD (range))	49.1	16.2 (16, 97)	48.8	16.2 (18, 93)	50.2	16.1 (16, 97)
Gender—female*	479	76.3	386	77.4	93	72.1
**Race/ethnicity**						
White/non-Hispanic	9	1.4	7	1.4	2	1.6
Black/non-Hispanic	193	30.7	150	30.1	43	33.3
Hispanic	346	55.1	281	56.3	65	50.4
Non-Hispanic	15	2.4	10	2.0	5	3.9
**Education level**						
No formal education	10	2	6	1.2	4	3.1
Less than high school	115	18	102	20.4	13	10.1
GED/High school diploma	235	37	197	39.5	38	29.5
Some/completed vocational/technical school	55	9	43	8.6	12	9.3
Some/completed college	94	15	80	16.0	14	10.9
Graduate/advanced degree	14	2	11	2.2	3	2.3
**Primary caregiver of a child < 6 years (yes vs no)**	99	15.8	79	15.8	20	15.5
**Housing status of alters compared to egos**						
Same household	72	11.46	67	13.4	5	3.9
Same housing development	175	27.87	118	23.7	57	44.2
Same neighborhood, different housing development	4	0.64	3	0.6	1	0.8
Same neighborhood, private residence	12	1.91	10	2.0	2	1.6
Another housing development in Boston, not in same neighborhood	40	6.37	30	6.0	10	7.8
Private residence in Boston, not in same neighborhood	161	25.64	135	27.1	26	20.2
Elsewhere*	156	24.84	132	26.5	24	18.6
Relational/tie-strength variables						
**Ego-alter relationship type**						
Family	342	54	301	60.3	41	31.8
Friend and others	283	45	197	39.5	86	66.7
**Closeness**						
Very close [5]	496	78.98	421	84.37	75	58.14
4	30	4.78	19	3.81	11	8.53
Somewhat close [3]	80	12.74	49	9.82	31	24.03
2	8	1.27	2	0.40	6	4.65
Not at all close [1]	6	0.96	4	0.80	2	1.55
**Frequency of contact**						
Daily	423	67.36	353	70.74	70	54.26
Weekly	143	22.77	104	20.84	39	30.23
Less than every week	50	7.96	38	7.62	12	9.3
Rarely or never	4	0.64	1	0.2	3	2.33
**Frequency of shared meals**						
Daily	124	19.75	108	21.64	16	12.4
Weekly	222	35.35	176	35.27	46	35.66
Less than every week	124	19.75	96	19.24	28	21.71
Rarely or never	151	24.04	115	23.05	36	27.91
**Years known**						
> 14 years	435	69.27	369	73.95	66	51.16
≤ 14 years	187	29.78	127	25.45	60	46.51
Alter-level behavior and health status						
**Sugar-sweetened beverage consumption frequency**						
Rarely or never	211	34	172	34.47	39	30.23
At least once a week, not every day	54	9	38	7.62	16	12.40
Once a day	129	21	104	20.84	25	19.38
Twice a day	70	11	59	11.82	11	8.53
Three times a day	51	8	42	8.42	9	6.98
Four times a day	34	5	28	6	6	5
Five or more times a day	30	5	23	4.61	7	5.43
**Sugar-sweetened food consumption frequency**						
Rarely or never	233	37	186	37.27	47	36.43
At least once a week, not every day	88	14	75	15.03	13	10.08
Once a day	107	17	83	16.63	24	18.60
Twice a day	77	12	60	12.02	17	13.18
Three times a day	33	5	28	5.61	5	3.88
Four times a day	22	4	18	3.61	4	3.10
Five or more times a day	20	3	17	3.41	3	2.33
**Smoking status**						
Daily smoker	80	13	55	11	25	19.38
Non-daily smoker	66	11	57	11	9	6.98
Former smoker	11	2	9	2	2	1.55
Never smoked	442	70	359	72	83	64.34
**Oral health status**						
Excellent	80	12.74	67	13.43	13	10.08
Very good	80	12.74	69	13.83	11	8.53
Good	285	45.38	223	44.69	62	48.06
Fair	97	15.45	79	15.83	18	13.95
Poor	34	5.41	25	5.01	9	6.98
**General health status**						
Excellent	68	11	56	11.22	12	9.30
Very good	70	11	55	11.02	15	11.63
Good	285	45	219	43.89	66	51.16
Fair	146	23	124	24.85	22	17.05
Poor	42	7	34	6.81	8	6.20
Ego-alter homophily variables						
**Sociodemographic homophily vs non-homophily**						
Age difference (years)	4.1	16.46 (− 40, 52)	4.3	16.53 (− 40, 52)	3.4	16.26 (− 39, 52)
Gender (yes vs no)	459	73.1	367	73.6	92	71.3
Hispanic ethnicity (yes vs no)	589	93.8	478	95.8	111	86.1
Education category (yes vs no)	352	56.1	304	60.9	48	37.2
Current primary caregiver of a child < 6 years (yes vs no)	483	76.9	392	78.6	91	70.5
**Behavior homophily vs non-homophily**						
Sugar-sweetened beverages (yes vs no)	327	52.1	263	52.7	64	49.6
Sugar-sweetened food (yes vs no)	305	48.6	251	50.3	54	41.9
Smoking status (yes vs no)	379	60.4	307	61.5	72	55.8
**Health status homophily vs non-homophily**						
Oral health status (yes vs no)	331	52.7	269	53.9	62	48.1
General health status (yes vs no)	374	59.6	297	59.5	77	59.7
**Network tie-type**						
Important matters	508	80.9	426	85.4	82	63.6
Shared meals	396	63.1	327	65.5	69	53.5
Public housing	235	37.4	177	35.5	58	45.0
**Multiplexity across reasons for relationship generation**						
No multiplexity	221	35.2	161	32.2	60	46.6
Important matters only	132	21.0	111	22.2	21	16.3
Shared meals only	18	2.9	12	2.4	6	4.7
Public housing only	71	11.3	38	7.6	33	25.6
**All reasons for relationship generation** *(Important matters, shared meals, and public housing, inclusive)*	118	18.8	101	20.2	17	13.2
**Shared meals and public housing**	24	3.8	16	3.2	8	6.2
**Important matters and public housing**	27	4.3	23	4.6	4	3.1
**Important matters and shared meals**	238	37.9	198	39.7	40	31.0

*SD* standard deviation

* Participants who selected ‘elsewhere’ were invited to write in their own response

**Table 3:** Bivariate associations of baseline characteristics (March 2019–2020) with retention of ego-alter ties (yes vs no) at follow-up (June 2021–August 2022) (*N* = 628)

	Retention of ego-alter ties at follow-up (yes vs no)			
	OR (95% CI)	*N*	AIC value	Significance
Network tie-type				
Important matters network only (yes vs no)	7.65 (3.72, 15.73)	628	542.6	***
Public housing network only (yes vs no)	0.55 (0.32, 0.95)	628	574	*
Shared meals network only (yes vs no)	1.89 (1.07, 3.32)	628	573.8	*
Multiplexity				
Multiplexity = 2 (vs 1)	1.9 (1.02, 3.55)	628	567.9	*
Multiplexity = 3 (vs 1)	4.43 (1.84, 10.67)			***
Relational/tie-strength variables				
Relationship type (family vs friends and others)	4.2 (2.37, 7.43)	625	545.3	***
Frequency of shared meals (daily vs less than daily)	2.03 (0.97, 4.23)	621	557.5	
Frequency of contact (daily vs less than daily)	2.63 (1.43, 4.84)	620	546.5	**
Closeness (very close vs not very close)	7.26 (3.37, 15.62)	620	530.8	***
Years known (> 14 years vs ≤ 14 years)	5.59 (2.86, 10.96)	622	534.2	***
Residence				
Same development vs same household	0.07 (0.02, 0.25)	620	536.4	***
Elsewhere* vs same household	0.23 (0.07, 0.81)	620	536.4	*
Ego-level individual variables				
**Ego-level demographics**				
Age (years)	0.99 (0.96, 1.02)	628	578.4	
Education (> high school vs ≤ high school)	0.86 (0.37, 1.98)	622	577.3	
Employment status (employed vs unemployed)	1.3 (0.61, 2.81)	628	578.2	
Hispanic ethnicity (yes vs no)	1.46 (0.64, 3.33)	628	577.9	
Current primary caregiver of a child < 6 years (yes vs no)	1.09 (0.38, 3.14)	628	578.7	
**Ego-level behavior**				
Sugar-sweetened beverages (daily vs < daily)	1.41 (0.61, 3.27)	628	578.1	
Sugar-sweetened food (daily vs < daily)	0.85 (0.35, 2.05)	628	578.6	
Smoking status (ever vs never)	0.47 (0.21, 1.02)	628	574.9	
**Ego-level health status**				
Oral health status (excellent/very good/good vs fair/poor)	1.1 (0.5, 2.43)	614	564	
General health status (excellent/very good/good vs fair/poor)	0.7 (0.32, 1.52)	628	577.9	
Alter-level individual variables				
**Alter-level demographics**				
Age (years)	1 (0.98, 1.01)	624	572.1	
Education (> high school vs ≤ high school)	0.69 (0.35, 1.36)	523	428.8	
Hispanic ethnicity (yes vs no)	1.02 (0.51, 2.03)	624	576.5	
Current primary caregiver of a child < 6 years (yes vs no)	0.86 (0.42, 1.78)	609	551.2	
**Alter-level behavior**				
Sugar-sweetened beverages (daily vs < daily)	1.11 (0.61, 2.02)	579	517.2	
Sugar-sweetened food (daily vs < daily)	0.67 (0.34, 1.3)	580	510.4	
Smoking status (ever vs never)	0.81 (0.44, 1.49)	599	547.7	
**Alter-level health status**				
Oral health status (excellent/very good/good vs fair/poor)	0.87 (0.41, 1.85)	576	508.5	
General health status (excellent/very good/good vs fair/poor)	0.53 (0.26, 1.07)	611	550.6	
Ego-alter homophily variables				
**Sociodemographic homophily vs non-homophily**				
Age difference (years)	1 (0.98, 1.02)	624	572.1	
Gender (yes vs no)	0.98 (0.54, 1.78)	621	573.3	
Hispanic ethnicity (yes vs no)	3.18 (1.23, 8.19)	628	573	*
Education category (yes vs no)	2.89 (1.68, 4.97)	622	562.2	***
Current primary caregiver of a child < 6 years (yes vs no)	2.14 (1.08, 4.32)	619	551.7	*
**Behavior homophily vs non-homophily**				
Sugar-sweetened beverages (yes vs no)	1 (0.57, 1.77)	623	564.2	
Sugar-sweetened food (yes vs no)	1.21 (0.67, 2.21)	623	563.8	
Smoking status (yes vs no)	1.13 (0.63, 2.03)	623	564	
**Health status homophily vs non-homophily**				
Oral health status (yes vs no)	1.34 (0.66, 2.73)	566	493.8	
General health status (yes vs no)	1.19 (0.61, 2.31)	611	553.6	

*OR* odds ratio, *CI* confidence interval, *AIC* Akaike information criterion

Variables modeled as index vs reference

* Significance levels: *p* < 0.05

** *p* < 0.01

*** *p* < 0.001

* Participants who selected “elsewhere” were invited to write in their own response

## Data Availability

Data will be provided upon reasonable request of the investigator/corresponding author.

## Appendix: “Staying in Place, Staying Connected: Exploring Tie-Retention to Inform Network-Based Health Interventions in Public Housing Communities”

**Table 4:** Baseline ego self-reported characteristics of those lost to follow up, March 2019–2020 (*N* = 85)

	*N*	%
**Age, years** *(mean, SD (range))*	47.4	15.4 (19, 87)
**Gender—female**	68	86.08
**Race/ethnicity**		
White/non-Hispanic	3	3.80
Black/non-Hispanic	37	46.84
Hispanic	40	50.63
Non-Hispanic	5	6.33
**Education level**		
No formal education	0	0.00
Less than high school	22	27.85
GED/High school diploma	31	39.24
Some/completed vocational/technical school	4	5.06
Some/completed college	23	29.11
Graduate/advanced degree	5	6.33
**Employment status**		
Full-time job	16	20.25
Part-time job	10	12.66
Occasional job	7	8.86
Unemployed	47	59.49
Retired	5	6.33
**Primary caregiver of a child < 6 years (yes vs no)**	17	21.52
**Ego-alter relationship type** *		
Family	142	54.83
Friend and others	117	45.17
**General health status**		
Excellent	6	7.59
Very good	11	13.92
Good	25	31.65
Fair	30	37.97
Poor	12	15.19
**Oral health status**		
Excellent	6	7.59
Very good	7	8.86
Good	31	39.24
Fair	23	29.11
Poor	14	17.72
**Sugar-sweetened beverage consumption**		
Rarely or never	16	20.25
At least once a week, not every day	9	11.39
Once a day	25	31.65
Twice a day	12	15.19
Three times a day	6	7.59
Four times a day	6	7.59
Five or more times a day	11	13.92
**Sugar-sweetened food consumption**		
Rarely or never	32	40.51
At least once a week, not every day	17	21.52
Once a day	12	15.19
Twice a day	9	11.39
Three times a day	5	6.33
Four times a day	1	1.27
Five or more times a day	3	3.80
**Smoking status**		
Daily smoker	13	16.46
Non-daily smoker	5	6.33
Former smoker	19	24.05
Never smoked	40	50.63

*SD* standard deviation

* Family: spouse (current or former), romantic partner (current or former), parent (mother or father), sibling (brother or sister), another relative (e.g., uncle), and friend: friend, acquaintance, coworker or associate, health professional, and religious leader/mentor

### Baseline name generator prompts

#### Important matters:

“Thinking about the past year, please tell me the names [first and last, or nickname] of the adults with whom you discussed important matters or from whom you sought advice. These may include people such as friends, family members, a spouse, or a significant other.”

#### Shared meals:

“Eating food, such as lunch or dinner, or drinking coffee, for example, can provide an opportunity to socialize and interact with others on a regular basis. Thinking over the past year, please tell me the names [first, last, and nickname] of the adults with whom you regularly interacted over food and/or beverages. These may be the same or different people than those you have already named.”

#### Public housing community:

“Now, thinking specifically about the people living in [housing development], please tell me the names [first, last, and nickname] of the adults living within [housing development] with whom you have interacted for any reason over the past year. For example, through friendships or through providing or accepting help such as rides or childcare. These may be the same or different people than those you have already named.”
